# Development and internal validation of a machine-learning-developed model for predicting 1-year mortality after fragility hip fracture

**DOI:** 10.1186/s12877-022-03152-x

**Published:** 2022-05-24

**Authors:** Nitchanant Kitcharanant, Pojchong Chotiyarnwong, Thiraphat Tanphiriyakun, Ekasame Vanitcharoenkul, Chantas Mahaisavariya, Wichian Boonyaprapa, Aasis Unnanuntana

**Affiliations:** 1 Department of Orthopaedics, Faculty of Medicine, Chiang Mai University, Chiang Mai, Thailand; 2 Department of Orthopaedic Surgery, Faculty of Medicine Siriraj Hospital, Mahidol University, 2 Wanglang Road, Bangkoknoi, 10700 Bangkok, Thailand; 3 Biomedical Informatics Center, Faculty of Medicine, Chiang Mai University, Chiang Mai, Thailand; 4 Golden Jubilee Medical Center, Faculty of Medicine Siriraj Hospital, Mahidol University, Bangkok, Thailand; 5 Siriraj Information Technology Department, Faculty of Medicine Siriraj Hospital, Mahidol University, Bangkok, Thailand

**Keywords:** Fragility hip fracture, Machine learning, Mortality prediction

## Abstract

**Background:**

Fragility hip fracture increases morbidity and mortality in older adult patients, especially within the first year. Identification of patients at high risk of death facilitates modification of associated perioperative factors that can reduce mortality. Various machine learning algorithms have been developed and are widely used in healthcare research, particularly for mortality prediction. This study aimed to develop and internally validate 7 machine learning models to predict 1-year mortality after fragility hip fracture.

**Methods:**

This retrospective study included patients with fragility hip fractures from a single center (Siriraj Hospital, Bangkok, Thailand) from July 2016 to October 2018. A total of 492 patients were enrolled. They were randomly categorized into a training group (344 cases, 70%) or a testing group (148 cases, 30%). Various machine learning techniques were used: the Gradient Boosting Classifier (GB), Random Forests Classifier (RF), Artificial Neural Network Classifier (ANN), Logistic Regression Classifier (LR), Naive Bayes Classifier (NB), Support Vector Machine Classifier (SVM), and K-Nearest Neighbors Classifier (KNN). All models were internally validated by evaluating their performance and the area under a receiver operating characteristic curve (AUC).

**Results:**

For the testing dataset, the accuracies were GB model = 0.93, RF model = 0.95, ANN model = 0.94, LR model = 0.91, NB model = 0.89, SVM model = 0.90, and KNN model = 0.90. All models achieved high AUCs that ranged between 0.81 and 0.99. The RF model also provided a negative predictive value of 0.96, a positive predictive value of 0.93, a specificity of 0.99, and a sensitivity of 0.68.

**Conclusions:**

Our machine learning approach facilitated the successful development of an accurate model to predict 1-year mortality after fragility hip fracture. Several machine learning algorithms (eg, Gradient Boosting and Random Forest) had the potential to provide high predictive performance based on the clinical parameters of each patient. The web application is available at www.hipprediction.com. External validation in a larger group of patients or in different hospital settings is warranted to evaluate the clinical utility of this tool.

**Trial registration:**

Thai Clinical Trials Registry (22 February 2021; reg. no. TCTR20210222003).

**Supplementary Information:**

The online version contains supplementary material available at 10.1186/s12877-022-03152-x.

## Introduction

As people live longer, the incidence of hip fracture is increasing, and it is estimated that there will be approximately 4.5 million cases per year worldwide by 2050 [1]. Osteoporotic hip fractures severely adversely affect the quality of life of older adults, resulting in substantially higher mortality and disability, and a markedly reduced quality of life [2]. Approximately 25% of older adults who sustain a hip fracture die within the first year [3], and this rate is 8 times higher than the mortality rate in the general population of older adults [4]. Advanced age, male sex, clinical comorbidities, cognitive impairment, type of fracture, choice of treatment, and ambulatory status have been proposed as potential prognostic factors for mortality after hip fracture [4–8]. However, since the prediction of death is complex and multifactorial, mortality cannot be predicted using a single variable. Identification of patients at high risk of death facilitates the modification of associated perioperative factors that can reduce mortality.

The recent development of machine learning techniques enables the development of healthcare-related outcome prediction tools that include perioperative parameters and clinical variables [9]. These techniques can evaluate real-world data, which often have complex nonlinear relationships between variables [10], and are capable of building models with performances that exceed those of conventional prediction methods [11]*.* Many studies have evaluated the performances of machine learning methods, particularly mortality prediction algorithms that have been developed for cardiac surgery [12], liver resection following colorectal cancer metastasis [13], traumatic head injury [14], critically ill influenza patients [15], and surgery for hepatocellular carcinoma [16]. The studies found that these algorithms had a better performance than conventional regression techniques. Developing a high-performance prediction model is beneficial as the goal of predicting mortality is to identify high-risk patients and provide clinicians with opportunities to consider what to do next to improve outcomes in these patients.

The high rate of 1-year mortality among older adults with fragility hip fracture suggests the need for a similar machine learning approach to predict death in this vulnerable population. Several models are already available. Artificial neural networks and logistic regression are well-known methods and have been extensively studied [17–22]. Support Vector Machine [23, 24], Naive Bayes [20, 24] and Random Forests [22–24] have also been used to predict mortality after hip fracture. However, there are other novel methods that demonstrate good performance with high accuracy in predicting death [15, 25, 26], such as Gradient Boosting, which have not yet been thoroughly explored for use in patients with hip fracture.

Since osteoporotic hip fracture occurs in a highly vulnerable population [27], an accurate prediction method would help clinicians identify patients who require special attention and additional services. This study aimed to develop and internally validate 7 machine learning models to predict 1-year mortality after fragility hip fracture in patients for whom a treatment decision (i.e. type of surgery or conservative treatment) had already been made. The models were the Gradient Boosting Classifier (GB), Random Forests Classifier (RF), Artificial Neural Network Classifier (ANN), Logistic Regression Classifier (LR), Naive Bayes Classifier (NB), Support Vector Machine Classifier (SVM), and K-Nearest Neighbors Classifier (KNN). We hypothesized that machine learning models could predict 1-year mortality after fragility hip fracture with high predictive performance.

## Methods

### Study design and population

This retrospective cohort study included patients with fragility hip fractures from a single center (the Department of Orthopedic Surgery, Faculty of Medicine Siriraj Hospital, Mahidol University, Bangkok, Thailand) from July 2016 to October 2018. The research protocol was approved by the Siriraj Institutional Review Board (approval number 122/2021), and the study was registered in the Thai Clinical Trials Registry on 22 February 2021 (registration number TCTR20210222003).

We used the International Classification of Diseases, Tenth revision (ICD-10) diagnosis codes S7200 (neck fracture of the femur), S7210 (intertrochanteric fracture of the femur), and S7220 (subtrochanteric fracture of the femur) to retrieve and review patient data from electronic medical records. Patients with fragility hip fracture were eligible for inclusion if they were aged 50 years or older and had a minimum follow-up period of 1 year or until death. The exclusion criteria were multiple fractures or fractures caused by cancer that had been confirmed by pathological study.

### Hip fracture treatment protocol

All patients with fragility hip fractures were attended by our fracture liaison service (FLS). The service provided a multidisciplinary care team consisting of orthopedic surgeons, metabolic bone disease specialists, anesthesiologists, geriatricians, physical therapists, physiatrists, and nurses. The team members provided post-fracture care programs and secondary-fracture prevention measures for the patients. Initially, the FLS team was alerted when patients with fragility hip fracture visited the emergency department. The acute pain management protocol was followed by anesthesiologists who specialized in pain medicine. The patients were then seen by the FLS team in an orthopedic ward. A geriatrician evaluated their medical condition and performed preoperative medical optimization. If surgery was decided, we operated as soon as the condition of each patient was suitable and an operating room was available. Conservative treatment was proposed for patients who already had a low probability of survival. A physical therapist started a rehabilitation program as early as possible to prevent complications from prolonged immobility. The multidisciplinary care team approach continued to play a key role in patient recovery, either after surgery or with conservative treatment. Physical therapists, in conjunction with psychiatrists in some cases, encouraged early mobilization for all patients. The physical therapists also assessed the risk of falling and planned appropriate home modification programs. FLS nurses facilitated the care process and reported each patient’s condition to other team members. As part of the secondary-fracture prevention program, metabolic bone specialists prescribed anti-osteoporosis medications, and osteoporosis education was given to patients and their families by orthopedic surgeons or nurses. The discharge planning process was carried out from the beginning of admission and was aimed at providing continuing care when patients were ready to leave the hospital. The entire care process was flexible and was adjusted according to the condition of each patient. FLS team meetings were scheduled every week for team members to review the status of each patient and discuss how to improve the care process. All patients were followed by telephone calls 3 and 12 months after discharge and yearly thereafter.

### Data collection

Demographic and clinical data were collected from electronic medical records. The data related to age; sex; body mass index (BMI), as stratified by the World Health Organization expert consultation for Asian populations [28]; Charlson Comorbidity Index (CCI) score; underlying diseases (presence of stage 4 or 5 chronic kidney disease [CKD], heart disease, lung disease, cerebrovascular accident [CVA], or dementia); type of fracture (femoral neck fracture, intertrochanteric fracture, or subtrochanteric fracture); type of treatment (conservative, dynamic hip screw fixation, multiple screw fixation, cephalomedullary nailing, hemiarthroplasty, or total hip arthroplasty); time to surgery; pre-injury ambulatory status (bedridden, indoor dependent, outdoor dependent, indoor independent, or outdoor independent); and walking assistive device (no ambulation, without assistive device, wheelchair, walker, quad cane, tripod cane, or single cane). We interviewed the patients or their relatives by telephone to assess the living status of the patients (1-year mortality after hip fracture). Because these factors had been shown to be essential predictors of mortality after hip fracture, they were used to develop a prediction model [4, 8, 29–32].

### Machine learning development process

#### Data preprocessing

**Fig. 1:**
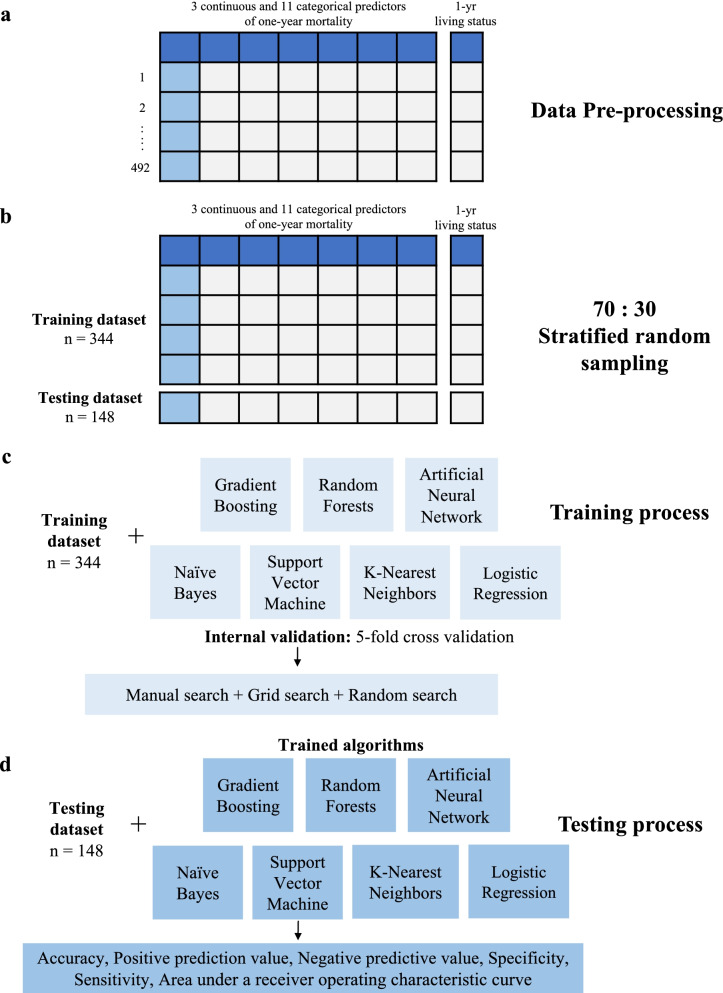
Machine learning development process (**a**) 3 continuous and 11 categorical predictors of one-year mortality were taken into the computational process. (**b**) A stratified random sampling technique was applied to split patients in a 70:30 ratio to a training dataset and a testing dataset. (**c**) Training dataset was used to identify the optimal hyperparameters which provided the highest accuracy in a fivefold internal cross-validation of each model. (**d**) The performance of all algorithms were evaluated with another, unseen, testing dataset

A de-identified dataset of 492 patients was enrolled in the study. Fifteen variables (3 continuous and 12 categorical) were collected. The continuous variables were age (integer), BMI (decimal number), and CCI score (integer). The categorical variables were sex, pre-injury status, pre-injury gait aid, CKD, heart disease, CVA, lung disease, dementia, diagnosis, type of treatment, time to surgery, and 1-year mortality after hip fracture. Using standard dummy coding, 3 continuous and 11 categorical predictors of one-year mortality were included in the computational process (Fig. 1a). There were no missing data in the dataset.

### Algorithm training and validation

We applied a stratified random sampling technique to split patients in a 70:30 ratio into a training dataset and a testing dataset (Fig. 1b). All variables were normalized to a scale of 0–1 to make the training process less sensitive to the scale of the variables. In this study, 7 machine-learning classifier algorithms [33] were used. They were the Gradient Boosting Classifier (GB), Random Forests Classifier (RF), Artificial Neural Network Classifier (ANN), Logistic Regression Classifier (LR), Naive Bayes Classifier (NB), Support Vector Machine Classifier (SVM), and K-Nearest Neighbors Classifier (KNN). Using the training dataset (344 patients), manual parameter tuning, grid search, and random search were conducted to identify the optimal hyperparameters [34] which provided the highest accuracy in a fivefold internal cross-validation of each model (Fig. 1c). Subsequently, we evaluated the performance of all algorithms by using another unseen testing dataset (148 patients; Fig. 1d). The confusion matrix and evaluation measures that were reported consisted of accuracy, positive predictive value, negative predictive value, specificity, sensitivity, calibration plots and area under the receiver operating characteristic curve (AUC). Calibration refers to how well the observed and the predicted outcomes match up. An optimal value of a slope and intercept for perfect calibration is 1 and 0, respectively. We evaluated the contribution of each characteristic to the prediction model using SHAP (Shapley values) [35, 36]. In these processes, the Python programming language (version 3.8.3; Python Software Foundation, Wilmington, DE, USA); and Scikit-Learn (version 0.24.2; Machine Learning library) [33] were used. All computational processes were performed in a Windows Server 2016 Datacenter (2.2 GHz × 4 virtual processors, with 15.9 GB of random-access memory).

### Statistical analysis

Comparisons were made of the baseline characteristics of the patients in the training and testing groups (Table 1), and of those who died and those who survived (Table 2). Continuous data were compared using Student’s *t*-test; the results are presented as mean plus/minus standard deviation. Categorical data were compared using the chi-squared test or Fisher’s exact test; these results are given as number and percentage. Data analyses were performed using PASW Statistics for Windows (version 18; SPSS Inc., Chicago, IL, USA). Accuracy, sensitivity, specificity, and positive and negative predictive values of all models were calculated and compared using the DTComPair package (https://cran.r-project.org/web/packages/DTComPair/DTComPair.pdf). We also compared the AUC of all models by performing permutation testing using the coin package (https://cran.r-project.org/web/packages/coin/index.html) in R software version 4.1.1 (http://www.r-project.org/). A two-tailed *P* value < 0.05 was considered statistically significant.

**Table 1:** Comparison of the demographic and clinical characteristics of all patients, and of those in the training and testing groups

Patient characteristics	Total (*N* = 492)	Testing (*n* = 148)	Training(*n* = 344)	*P* value
Age (years), mean ± SD	78.4 ± 9.8	78.0 ± 10.1	78.6 ± 9.7	0.511
Female sex, *n* (%)	355 (72.2%)	104 (70.3%)	251 (73.0%)	0.584
Body mass index (kg/m.^2^), mean ± SD	22.3 ± 3.9	22.2 ± 4.1	22.4 ± 3.9	0.634
Charlson comorbidity index (CCI) score, *n* (%)				
- < 3	39 (7.9%)	13 (8.8%)	26 (7.6%)	0.716
- ≥ 3	453 (92.1%)	135 (91.2%)	318 (92.4%)	
Pre-injury ambulatory status, *n* (%)				
- Bedridden	15 (3.0%)	7 (4.7%)	8 (2.3%)	0.556
- Indoor dependent	43 (8.7%)	10 (6.8%)	33 (9.6%)	
- Outdoor dependent	16 (3.3%)	5 (3.4%)	11 (3.2%)	
- Indoor independent	145 (29.5%)	45 (30.4%)	100 (29.1%)	
Assistive device, *n* (%)				
- No ambulation	15 (3.0%)	7 (4.7%)	8 (2.3%)	0.839
- Without assistive device	259 (52.6%)	76 (51.4%)	183 (53.2%)	
- Wheelchair	10 (2.0%)	2 (1.4%)	8 (2.3%)	
- Walker	91 (18.5%)	28 (18.9%)	63 (18.3%)	
- Quad cane	3 (0.6%)	1 (0.7%)	2 (0.6%)	
- Tripod cane	27 (5.5%)	9 (6.1%)	18 (5.2%)	
- Single cane	27 (5.5%)	25 (16.9%)	62 (18.0%)	
Type of fracture, *n* (%)				
- Femoral neck fracture	248 (50.4%)	77 (52.0%)	171 (49.7%)	0.883
- Intertrochanteric fracture	241 (49.0%)	70 (47.3%)	171 (49.7%)	
- Subtrochanteric fracture	3 (0.6%)	1 (0.7%)	2 (0.6%)	
Treatment, *n* (%)				
- Conservative treatment	32 (6.5%)	11 (7.4%)	21 (6.1%)	0.791
- Dynamic hip screw	36 (7.3%)	12 (8.1%)	24 (7.0%)	
- Cephalomedullary nailing	202 (41.1%)	56 (37.8%)	146 (42.4%)	
- Multiple screw fixation	19 (3.9%)	8 (5.4%)	11 (3.2%)	
- Hemiarthroplasty	195 (39.6%)	59 (39.9%)	136 (39.5%)	
- Total hip arthroplasty	8 (1.6%)	2 (1.4%)	6 (1.7%)	
Comorbidities, *n* (%)				
- Chronic kidney disease stage 4 or severe	130 (26.4%)	37 (25.0%)	93 (27.0%)	0.658
- Heart disease	123 (25.0%)	44 (29.7%)	79 (23.0%)	0.114
- Cerebrovascular accident	103 (20.9%)	35 (23.6%)	68 (19.8%)	0.336
- Lung disease	34 (6.9%)	13 (8.8%)	21 (6.1%)	0.332
- Dementia	81 (16.5%)	16 (10.8%)	65 (18.9%)	0.033
Time to surgery *n* (%)				
- ≤ 48 h	233 (50.7%)	69 (50.4%)	164 (50.8%)	1.000
- > 48 h	227 (49.3%)	68 (49.6%)	159 (49.2%)	
Death, *n* (%)	62 (12.6%)	19 (12.8%)	43 (12.5%)	1.000

A *P* value < 0.05 indicates statistical significance

Abbreviation: *SD* Standard deviation

**Table 2:** Comparison of the demographic and clinical characteristics of patients who died and those who survived

Patient characteristics	Deceased (*n* = 62)	Survived (*n* = 430)	*P* value
Age (years), mean ± SD	81.5 ± 8.5	77.9 ± 9.9	***0.007***
Male sex, n (%)	25 (40.3%)	112 (26.0%)	***0.023***
Body mass index (kg/m.^2^), mean ± SD	21.8 ± 4.2	22.4 ± 3.9	0.123
Charlson comorbidity index (CCI), *n* (%)			
- < 3	0 (0.0%)	39 (9.1%)	***0.009***
- ≥ 3	62 (100.0%)	391 (90.9%)	
Pre-injury ambulatory status, *n* (%)			
- Bedridden	1 (1.6%)	14 (3.3%)	***0.021***
- Indoor dependent	10 (16.1%)	33 (7.7%)	
- Outdoor dependent	1 (1.6%)	15 (3.5%)	
- Indoor independent	24 (38.7%)	121 (28.1%)	
- Outdoor independent	26 (42.0%)	247 (57.4%)	
Assistive device, *n* (%)			
- No ambulation	1 (1.6%)	14 (3.3%)	0.421
- Without assistive device	26 (41.9%)	233 (54.2%)	
- Wheelchair	2 (3.2%)	8 (1.9%)	
- Walker	17 (27.4%)	74 (17.2%)	
- Quad cane	0 (0.0%)	3 (0.7%)	
- Tripod cane	4 (6.5%)	23 (5.3%)	
- Single cane	12 (19.4%)	75 (17.4%)	
Type of fracture, *n* (%)			
- Femoral neck fracture	20 (32.3%)	228 (53.0%)	***0.001***
- Intertrochanteric fracture	42 (67.7%)	199 (46.3%)	
- Subtrochanteric fracture	0 (0.0%)	3 (0.7%)	
Treatment, *n* (%)			
- Conservative treatment	13 (21.0%)	19 (4.4%)	***0.022***
- Dynamic hip screw	5 (8.1%)	31 (7.2%)	
- Cephalomedullary nailing	27 (43.5%)	175 (40.7%)	
- Multiple screw fixation	2 (3.2%)	17 (4.0%)	
- Hemiarthroplasty	15 (24.2%)	180 (41.8%)	
- Total hip arthroplasty	0 (0.0%)	8 (1.9%)	
Time to surgery *n* (%)			
- ≤ 48 h	19 (38.8%)	214 (52.1%)	0.096
- > 48 h	30 (61.2%)	197 (47.9%)	
Comorbidities, *n* (%)			
- Chronic kidney disease stage 4 or severe	44 (71.0%)	86 (20.0%)	** < *0.001***
- Heart disease	41 (66.1%)	82 (19.1%)	** < *0.001***
- Cerebrovascular accident	24 (38.7%)	79 (18.4%)	***0.001***
- Lung disease	17 (27.4%)	17 (4.0%)	** < *0.001***
- Dementia	27 (43.5%)	54 (12.6%)	** < *0.001***

A *P* value < 0.05 indicates statistical significance

Abbreviation: *SD* Standard deviation

## Results

From July 2016 to October 2018, 498 patients with a hip fracture were admitted to our institution. Six patients had multiple fractures and were excluded from our study, leaving 492 for final analysis. Through telephone interviews, we were able to obtain the living status of all patients 1 year after the respective fragility hip fractures.

### Baseline characteristics

The mean age of the study participants was 78.4 years (range, 50–101). Of the 492 enrolled patients, 72.2% were women and 27.8% were men (Table 1). Four hundred fifty-three patients (92.1%) had a CCI score ≥ 3, and 259 (52.6%) walked without an assistive device. The majority (55.5%) could ambulate outdoors independently before the hip fracture. Four hundred and sixty patients (93.5%) underwent operative treatment. During the study period, 50.7% of the patients were able to undergo surgery within 48 h of admission. The median length of stay was 11 days (interquartile range 8–17 days). The median time from admission to surgery was 2 days (interquartile range 1–4 days). The 1-year mortality rate after hip fracture was 12.6%.

We randomly assigned 344 and 148 patients to the training and testing datasets, respectively. There were no significant differences in the patient characteristics of the 2 datasets. A comparison of the characteristics of the patients who survived and those who died is presented in Table 2. It revealed that the deceased group was significantly older (*P* = 0.007), had a significantly higher proportion of male patients (*P* = 0.023), and had significantly higher prevalences of all 5 evaluated comorbidities (stage 4 or 5 CKD, heart disease, CVA, lung disease, and dementia; all *P* = 0.001 or *P* < 0.001). CCI score, pre-injury ambulatory status, type of fracture, and treatment were also significantly different.

### Machine-learning performance comparisons

**Fig. 2:**
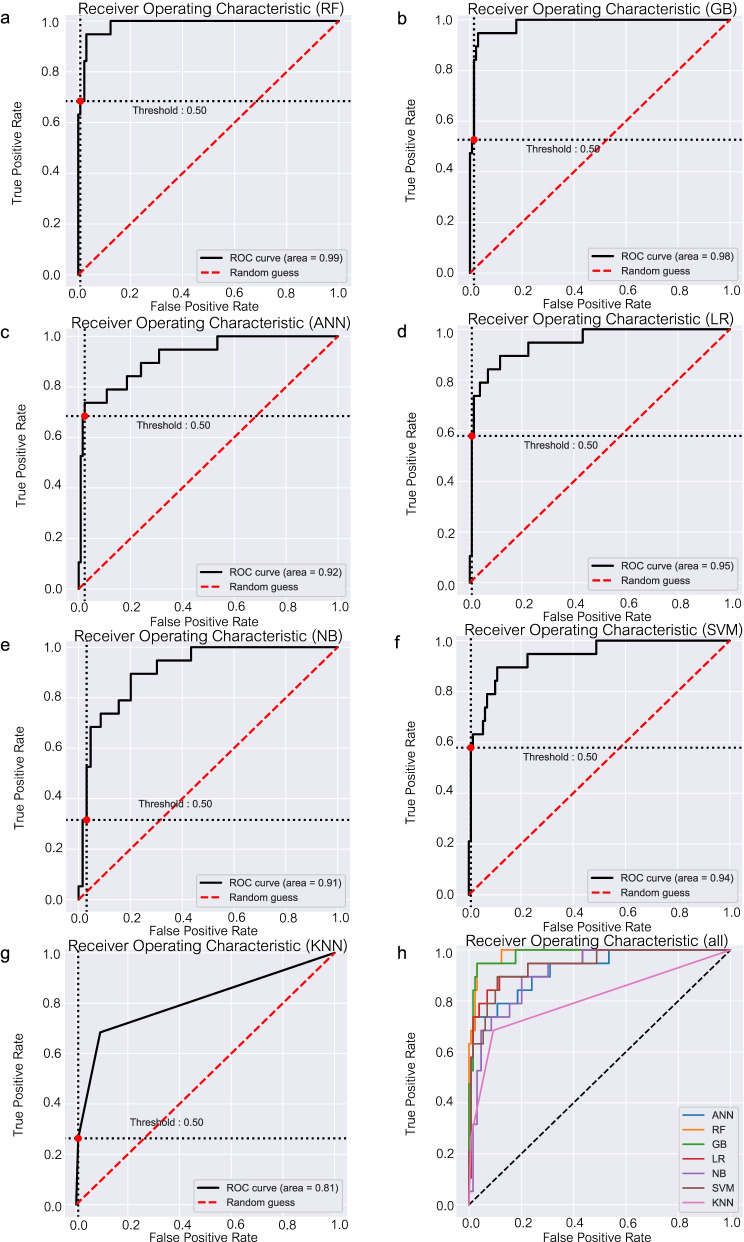
Receiver-operating characteristic curve (ROC) of **(a)** Random Forests algorithm (RF); **(b)** Gradient Boosting algorithm (GB); **(c)** Artificial Neural Network algorithm (ANN); **(d)** Logistic Regression algorithm (LR); **(e)** Naive Bayes algorithm (NB); **(f)** Support Vector Machine algorithm (SVM); **(g)** K-Nearest Neighbors algorithm (KNN); and **(h)** all algorithms

We used a dataset of 344 patients to train 7 machine learning models to predict 1-year mortality after fragility hip fracture. The performances of the 7 algorithms are detailed in Table 3. For the training dataset, the accuracies were GB model = 1.00, RF model = 0.97, ANN model = 0.99, LR model = 0.94, NB model = 0.90, SVM model = 0.94, and KNN model = 0.94. As to the testing dataset, all models achieved high AUCs (between 0.81 and 0.99; Fig. 2). The RF model provided high predictive performance, with an accuracy of 0.95, a positive predictive value of 0.93, and a sensitivity of 0.68. There were significant differences between the AUC of RF model and the ANN, LR, NB, SVM, KNN models (0.99 vs 0.92 vs 0.95 vs 0.91 vs 0.94 vs 0.81, respectively). The calibration of all models show intercepts ranging from -0.09 to 0.35 and slopes ranging from 0.55 to 1.32 (see Supplementary file 1). The calibration plot of the RF model was well calibrated with slope and intercept close to optimal value.

**Table 3:** Comparison of the performance of each model, by confusion matrix and evaluation measures

Models	Testing dataset													
	**Predict deceased**	**Predict survived**	**Accuracy [95% CI]**	***P* value** **(vs RF model)**	**Positive predictive value [95% CI]**	***P* value** **(vs RF model)**	**Negative predictive value [95% CI]**	***P* value** **(vs RF model)**	**Specificity [95% CI]**	***P* value** **(vs RF model)**	**Sensitivity [95% CI]**	***P* value** **(vs RF model)**	**AUC [95% CI]**	***P* value** **(vs RF model)**
**Random Forests (RF)**														
Deceased	13	6	0.95 [0.91–0.98]		0.93 [0.66–1.00]		0.96 [0.91–0.98]		0.99 [0.96–1.00]		0.68 [0.43–0.87]		0.99 [0.95–1.00]	
Survived	1	128												
**Gradient Boosting (GB)**														
Deceased	11	8	0.93 [0.88–0.97]	0.11	0.85 [0.55–0.98]	0.49	0.94 [0.89–0.97]	0.31	0.98 [0.95–1.00]	0.56	0.58 [0.33–0.80]	0.32	0.98 [0.94–1.00]	0.47
Survived	2	127												
**Artificial Neural Network (ANN)**														
Deceased	13	6	0.94 [0.89–0.97]	0.5	0.81 [0.54–0.96]	0.32	0.95 [0.90–0.98]	0.95	0.98 [0.93–1.00]	0.32	0.68 [0.43–0.87]	1	0.92 [0.83–1.00]	***0.01***
Survived	3	126												
**Logistic Regression (LR)**														
Deceased	7	12	0.91 [0.85–0.95]	***0.01***	0.88 [0.47–1.00]	0.69	0.91 [0.86–0.95]	***0.03***	0.99 [0.96–1.00]	1	0.37 [0.16–0.62]	***0.03***	0.95 [0.88–1.00]	***0.02***
Survived	1	128												
**Naive Bayes (NB)**														
Deceased	7	12	0.89 [0.83–0.94]	***0.02***	0.64 [0.31–0.89]	***0.01***	0.91 [0.85–0.95]	***0.03***	0.97 [0.92–0.99]	0.18	0.37 [0.16–0.62]	***0.03***	0.91 [0.82–1.00]	***0.02***
Survived	4	125												
**Support Vector Machine (SVM)**														
Deceased	5	14	0.90 [0.84–0.94]	***0.01***	0.83 [0.36–1.00]	0.58	0.90 [0.84–0.95]	***0.02***	0.99 [0.96–1.00]	1	0.26 [0.09–0.51]	***0.02***	0.94 [0.86–1.00]	***0.02***
Survived	1	128												
**K-Nearest Neighbors (KNN)**														
Deceased	5	14	0.90 [0.84–0.94]	***0.01***	0.83 [0.36–1.00]	0.58	0.90 [0.84–0.95]	***0.02***	0.99 [0.96–1.00]	1	0.26 [0.09–0.51]	***0.02***	0.81 [0.68–0.93]	***0.01***
Survived	1	128												

A *P* value < 0.05 indicates statistical significance

Abbreviation: *CI* Confidence interval

### Machine-learning-model selection

The RF model demonstrated high accuracy, positive predictive value, and sensitivity. Due to our screening-test study design, we mainly focused on sensitivity to detect at-risk patients who might encounter mortality 1 year after their fracture. The sensitivity of RF model were not significant different from GB and ANN model. However, the RF model had higher AUC than the ANN model. The RF model also had good calibration. Accordingly, the RF algorithm was selected for model construction.

### Analysis of clinical variable contribution

**Fig. 3:**
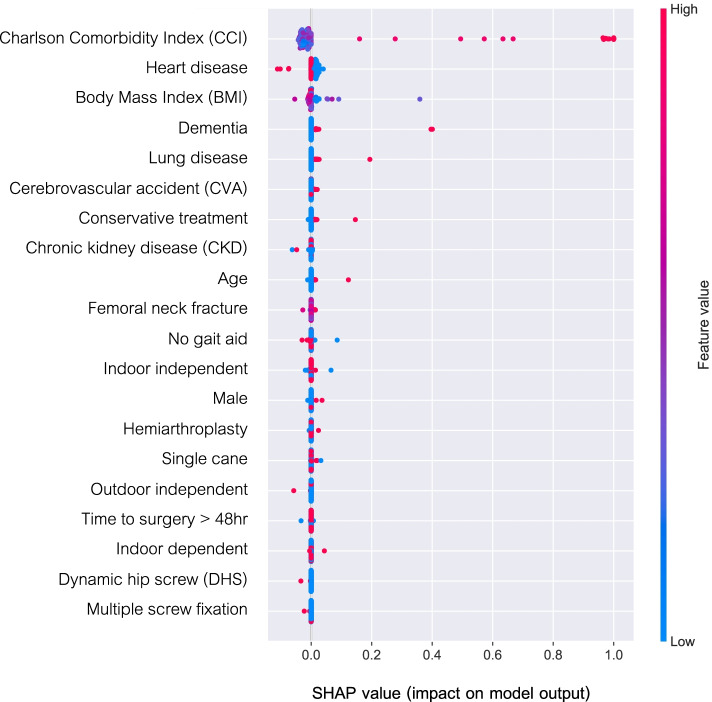
Characteristics of the selected model (Random Forests model): SHAP Value summary graph of top-20 variables and their impact on the prediction

Figure 3 illustrates the impact of each characteristic on the entire dataset prediction by the trained RF algorithm. The 5 most influential clinical characteristics were CCI score, heart disease, BMI, dementia, and lung disease. The best-tuned hyperparameters for the RF obtained from hyperparameter optimization were max_depth = 60, max_features = ‘sqrt', min_samples_leaf = 4, min_samples_split = 5, n_estimators = 400, and random_state = 8. The best-tuned hyperparameters for all models are listed in Table 4. The receiver operating characteristic curve of the Random Forests algorithm is shown in Fig. 2a.

**Table 4:** The best-tuned hyperparameters for each model

Classifier models	Hyperparameters
Gradient Boosting	max_depth = 10, max_features = 'sqrt', min_samples_split = 50, n_estimators = 800, random_state = 8, learning_rate = 0.5, subsample = 0.5
Random Forests	max_depth = 60, max_features = 'sqrt', min_samples_split = 5, min_samples_leaf = 4, n_estimators = 400, random_state = 8
Artificial Neural Network	activation = 'identity', alpha = 0.0001, batch_size = 'auto', hidden_layer_sizes = 7, learning_rate = 'adaptive', learning_rate_init = 0.001, max_iter = 500, solver = 'lbfgs'
Logistic Regression	C = 0.4, multi_class = 'multinomial', random_state = 8, solver = 'saga'
Naive Bayes	alpha = 1.0, fit_prior = True, class_prior = None
Support Vector Machine	C = 0.1, degree = 4, kernel = 'poly', probability = True, random_state = 8
K-Nearest Neighbors	n_neighbors = 3

### Machine learning model application

The trained RF algorithm subsequently used demographic and clinical information to construct a predictive model to estimate the probability of 1-year mortality of patients. A programming interface was developed to allow healthcare providers to access the application at www.hipprediction.com. By entering details of key characteristics into the prediction model, it was able to generate the probability of 1-year mortality of individual patients with fragility hip fractures. The characteristics were age; sex; BMI; pre-injury ambulatory status; assistive device usage; CCI score; type of fracture; type of operation; time to surgery; and the presence of CKD, heart disease, CVA, lung disease, and dementia.

## Discussion

Patients with osteoporotic hip fracture are likely to suffer higher morbidity and mortality than non-fracture patients within the same age group [4, 37]. Adverse events after hip fracture can occur during hospitalization and the post-discharge period. They include events such as infection [38], heart failure [38], and thromboembolism [39]. As these events can lead to death in high-risk patients [40, 41], an attempt to identify those at risk of complications after hip fracture is a primary objective to reduce mortality. In patients predicted to be at risk for poor outcomes, interventions can be initiated to prevent complications and reduce the likelihood of death. Among the possible interventions, two are paramount. The first requires full and honest communication with the patient and family about the planned treatment and risks. The second requires prompt clinical decision-making by doctors, families, and caregivers to ensure that scarce resources, such as an intensive care unit, are effectively allocated, and that the need for additional services is determined and actioned. Examples of these services are intensive monitoring and optimization of patients’ medical problems, additional home visits and family nursing support, and a personalized exercise program. In this study, we evaluated machine learning methods to develop a model that would predict 1-year mortality after a fragility hip fracture. Our results showed that the tool we designed had high mortality-prediction accuracy.

Our experiment carried out a 3-step, general, machine learning approach: data preprocessing, algorithm training, and algorithm testing with an unseen dataset. We searched for the best performance of each algorithm by fivefold cross-validation using manual search and automated hyperparameter optimization with grid search and random search [34]. After comparing all algorithms, we selected the RF model. It provided the highest performance in predicting 1-year mortality, indicated by its highest sensitivity in detecting high-risk patients (Table 3).

GB and RF models are tree-based methods that gather the results from individual trees. The difference between the 2 models is how the trees build up and how the results are collected [42, 43]. The GB model adds each tree up sequentially and allows self-correction from the error at each step to improve the model, while the RF model builds all trees up simultaneously. GB collects the results during the whole process from start to finish, while RF sums up and averages the results when the process is finished. Theoretically, GB usually takes a longer time to train, but it can provide better performance than RF if the parameters are carefully tuned. GB and RF have also demonstrated their high performance in predictive modeling of health outcomes [15, 25, 26, 44].

The ANN models used in this study were standard feed-forward, multilayer perceptrons with back-propagation neural networks trained using a supervised training algorithm [45]. Each of the ANN models consists of 3 layers: 1 input layer, 1 hidden layer, and 1 output layer. The neural network takes input variables, which are then passed through the layer of hidden neurons to the output layer. The ANN model is a flexible system that allows complex modeling of nonlinear relationships. It is not adversely influenced by the interconnection of multiple variables, which is the case with patients with a hip fracture [46]. Moreover, the ANN model can automatically adjust the weight in the network and self-correct, which produces a better prediction accuracy [47].

In contrast, LR is commonly used to predict the probability of occurrence of an event. It assumes that the outcome has a linear relationship with the variables [48]. The LR method predefines the association among the predictors in a linear manner, which gives it the ability to explain the degree of causal relationship for each variable [17, 49, 50]. However, if there is interplay between or among the factors or a nonlinear relationship exists, the LR model may be a less appropriate modeling option [51] for our dataset.

The NB algorithm is a classification technique that applies Bayes’ theorem by assuming that each variable is independent of each other [52]. This assumption makes the learning phase easier and simple to implement. However, the NB algorithm might be inaccurate in scenarios with increased bias for nonlinear problems. NB might also have a better performance in datasets that have a small sample size [53].

SVM utilizes a geometrical relationship between variables and predicts outcomes by identifying the boundary (or hyperplane) between the data of 2 classes and separating them. It has a good performance in distinguishing between 2 classes, provides flexibility for both linear and nonlinear problems, and has a low risk of overfitting from its regularization feature [54, 55]. However, interpretation of the model is often difficult [56, 57].

The KNN creates decision boundaries to separate different classes [58]. Its advantage is that it is simple to implement and easy to understand. It also utilizes a memory-based approach and is capable of being quickly trained with a new dataset. However, if the K value is not appropriately chosen, the model has a high risk of overfitting [59, 60].

Although most machine learning approaches offer flexibility in solving sophisticated connections between variables and outcomes, interpretation problems can arise and present a challenge to implementation [61]. One way to explore how each predictor affects the outcome of interest is to apply the Shapley (SHAP) values to rank the predictors according to their contribution to a model [35, 36]. In Fig. 3, the SHAP-value graph illustrates the value of the top 20 variables and their impact on the predictions of the RF model. It explains why a high CCI score increased the predicted 1-year mortality. CCI scores are calculated by considering multiple comorbidities of patients, and the scoring system has been validated to predict 1-year mortality [62]. CCI scores have also been reported to be related to reduced survival in women after suffering hip fracture [63]. As a result, the CCI score became the most influential characteristic in our model. Furthermore, dementia, lung disease, heart disease, and BMI were found to be important predictive factors for 1-year mortality. When we compared the characteristics of the patients who died and those who survived, all characteristics differed significantly, other than BMI and gait aid. All of these statistically different characteristics had also been reported to be significantly associated with post-fragility fracture mortality by other studies. The significant differences we found in almost all factors support those previous findings and emphasize the essential predictive value of the factors. Advanced age and male sex were significant predictors of mortality [29–31]. A difference in mortality was observed between operative and nonoperative treatments [4]. Multiple comorbidities and preoperative mobility were also reported to be associated with death after hip fracture [8, 32]. However, despite a trend toward an improvement in the mortality rate and increased knowledge and awareness of these factors, a recent systematic review found that the mortality rate within 1 year of a hip fracture remains as high as 22% [64]. The development of a tool that can combine multiple variables into a single prediction model would be of great utility to clinicians. With advances in the machine learning approach, we can train and test models with datasets to recognize patterns that would otherwise be hidden in complex relationships between variables [65].

Various machine learning models for mortality prediction in hip fracture patients have been proposed [17–21, 23, 24, 66]. Unlike most previous studies, we investigated only hip fractures from low-energy trauma, and we did not exclude patients aged less than 65 years because osteoporotic hip fracture can occur at the age of 50 [67]. We also included variables, such as different kinds of hip fracture (ie, neck, intertrochanteric, and subtrochanteric fracture of the femur) and whether the patient received operative or nonoperative treatment. Although most hip fractures are currently managed operatively [68], there is a proportion of patients who are managed nonoperatively (eg, hip fracture patients whose pre-injury status was nonambulatory, and patients with an already low probability of survival). A recent systematic review showed that nonoperative treatment was associated with higher rates of morbidity and mortality [69], which further emphasizes the need for special attention to prevent complications in this group of patients.

Our machine-learning-developed model serves as a screening tool for the identification of high-risk patients and provides information that aids clinical decision-making. For instance, a prediction of death within 1 year would encourage physicians to develop an intensive treatment plan and prepare the resources needed for high-risk patients. These actions and interventions would be expected to reduce complications and improve patient survival. To identify at-risk patients with high accuracy, machine learning algorithms (eg, Gradient Boosting and Random Forests) can be used to develop models with acceptable predictive performance. The prediction tool can also be used to counsel patients and caregivers and encourage them to comply with the medical actions and interventions considered necessary.

### Limitations

This study is not without limitations. Like other hospitals, our center admits patients directly from their homes after suffering a hip fracture. However, being a tertiary care center, our hospital also accepts patients with very high-risk comorbidities who have been transferred from primary hospitals that do not have the resources needed for their treatment. This could lead to a selection bias. First, patients with severe comorbidities who are transferred to our hospital have a higher risk of mortality than the general population. In addition, any increase in the duration before surgery resulting from delays in transferring the patients to our hospital only exacerbates the risk of mortality [70]. However, we did not incorporate the time gap between fracture and hospital admission into our model and this could contribute to another potential limitation.

Second, our data were drawn from only a single center with a relatively small sample. This may have resulted in overfitting of the models. Therefore, external validation is essential to confirm the predictive ability of our 1-year mortality prediction tool. It is important to note that each center has different protocols for treating patients with hip fractures. This may also help explain the heterogeneity in the outcomes of earlier studies.

Finally, it should be noted that there are other factors that influence outcomes that were not included in our study, such as complications in the hospital and after discharge. These may prove to have some value in predicting mortality after hip fracture.

## Conclusions

Our machine learning approach facilitated the successful development of an accurate model to predict 1-year mortality after fragility hip fracture. Several machine learning algorithms (eg, Gradient Boosting and Random Forest) had the potential to provide high predictive performance, based on the clinical parameters of each patient. The 5 most influential clinical variables in the prediction model were the CCI score, heart disease, BMI, dementia, and lung disease. The web application is available at www.hipprediction.com. External validation in a larger group of patients or in different hospital settings is warranted to evaluate the clinical utility of this tool.

## Supplementary Information

**Additional file 1.** 

## Data Availability

The datasets used and/or analyzed during the current study are available from the corresponding author on reasonable request.

## Abbreviations

ANN: Artificial Neural Network
AUC: Area under the receiver operating characteristic curves
BMI: Body mass index
CCI: Charlson Comorbidity Index
CI: Confidence interval
CKD: Chronic kidney disease
CVA: Cerebral vascular accident
FLS: Fracture Liaison Service
GB: Gradient Boosting
KNN: K-Nearest Neighbors
LR: Logistic Regression
NB: Naive Bayes
RF: Random Forests
SVM: Support Vector Machine

## References

[1] Aubrun F (2011). Hip fracture surgery in the elderly patient: epidemiological data and risk factors. Ann Fr Anesth Reanim.

[2] Center JR, Nguyen TV, Schneider D, Sambrook PN, Eisman JA (1999). Mortality after all major types of osteoporotic fracture in men and women: an observational study. Lancet.

[3] Hannan EL, Magaziner J, Wang JJ, Eastwood EA, Silberzweig SB, Gilbert M (2001). Mortality and locomotion 6 months after hospitalization for hip fracture: risk factors and risk-adjusted hospital outcomes. JAMA.

[4] Vaseenon T, Luevitoonvechkij S, Wongtriratanachai P, Rojanasthien S (2010). Long-term mortality after osteoporotic hip fracture in Chiang Mai. Thailand J Clin Densitom.

[5] Pioli G, Lauretani F, Davoli ML, Martini E, Frondini C, Pellicciotti F (2012). Older people with hip fracture and IADL disability require earlier surgery. J Gerontol A Biol Sci Med Sci.

[6] Mariconda M, Costa GG, Cerbasi S, Recano P, Aitanti E, Gambacorta M, et al. The determinants of mortality and morbidity during the year following fracture of the hip: a prospective study. Bone Joint J. 2015;97–b:383–90. 10.1302/0301-620x.97b3.34504. 10.1302/0301-620X.97B3.34504 25737523

[7] Kim SM, Moon YW, Lim SJ, Yoon BK, Min YK, Lee DY (2012). Prediction of survival, second fracture, and functional recovery following the first hip fracture surgery in elderly patients. Bone.

[8] Smith T, Pelpola K, Ball M, Ong A, Myint PK (2014). Pre-operative indicators for mortality following hip fracture surgery: a systematic review and meta-analysis. Age Ageing.

[9] Doupe P, Faghmous J, Basu S (2019). Machine Learning for Health Services Researchers. Value Health.

[10] Hastie T, Tibshirani R, Friedman J (2009). The elements of statistical learning: data mining, inference, and prediction.

[11] Schwalbe N, Wahl B (2020). Artificial intelligence and the future of global health. Lancet.

[12] Nilsson J, Ohlsson M, Thulin L, Höglund P, Nashef SA, Brandt J (2006). Risk factor identification and mortality prediction in cardiac surgery using artificial neural networks. J Thorac Cardiovasc Surg.

[13] Spelt L, Nilsson J, Andersson R, Andersson B (2013). Artificial neural networks—a method for prediction of survival following liver resection for colorectal cancer metastases. Eur J Surg Oncol.

[14] Lang EW, Pitts LH, Damron SL, Rutledge R (1997). Outcome after severe head injury: an analysis of prediction based upon comparison of neural network versus logistic regression analysis. Neurol Res.

[15] Hu CA, Chen CM, Fang YC, Liang SJ, Wang HC, Fang WF (2020). Using a machine learning approach to predict mortality in critically ill influenza patients: a cross-sectional retrospective multicentre study in Taiwan. BMJ Open.

[16] Shi HY, Lee KT, Wang JJ, Sun DP, Lee HH, Chiu CC (2012). Artificial neural network model for predicting 5-year mortality after surgery for hepatocellular carcinoma: a nationwide study. J Gastrointest Surg.

[17] Lin CC, Ou YK, Chen SH, Liu YC, Lin J (2010). Comparison of artificial neural network and logistic regression models for predicting mortality in elderly patients with hip fracture. Injury.

[18] Shi L, Wang XC, Wang YS (2013). Artificial neural network models for predicting 1-year mortality in elderly patients with intertrochanteric fractures in China. Braz J Med Biol Res.

[19] Chen CY, Chen YF, Chen HY, Hung CT, Shi HY (2020). Artificial Neural Network and Cox Regression Models for Predicting Mortality after Hip Fracture Surgery: A Population-Based Comparison. Medicina (Kaunas).

[20] DeBaun MR, Chavez G, Fithian A, Oladeji K, Van Rysselberghe N, Goodnough LH (2020). Artificial Neural Networks Predict 30-Day Mortality After Hip Fracture: Insights From Machine Learning. J Am Acad Orthop Surg.

[21] Cary MP, Zhuang F, Draelos RL, Pan W, Amarasekara S, Douthit BJ (2021). Machine Learning Algorithms to Predict Mortality and Allocate Palliative Care for Older Patients With Hip Fracture. J Am Med Dir Assoc.

[22] Li Y, Chen M, Lv H, Yin P, Zhang L, Tang P (2021). A novel machine-learning algorithm for predicting mortality risk after hip fracture surgery. Injury.

[23] Lo C-L, Yang Y-H, Hsu C-J, Chen C-Y, Huang W-C, Tang P-L (2020). Development of a Mortality Risk Model in Elderly Hip Fracture Patients by Different Analytical Approaches. Appl Sci.

[24] Forssten MP, Bass GA, Ismail AM, Mohseni S, Cao Y (2021). Predicting 1-Year Mortality after Hip Fracture Surgery: An Evaluation of Multiple Machine Learning Approaches. J Pers Med.

[25] Yao RQ, Jin X, Wang GW, Yu Y, Wu GS, Zhu YB (2020). A Machine Learning-Based Prediction of Hospital Mortality in Patients With Postoperative Sepsis. Front Med (Lausanne).

[26] Cowling TE, Cromwell DA, Bellot A, Sharples LD, van der Meulen J (2021). Logistic regression and machine learning predicted patient mortality from large sets of diagnosis codes comparably. J Clin Epidemiol.

[27] Ko FC, Morrison RS (2014). Hip fracture: a trigger for palliative care in vulnerable older adults. JAMA Intern Med.

[28] WHO Expert Consultation (2004). Appropriate body-mass index for Asian populations and its implications for policy and intervention strategies. Lancet.

[29] White BL, Fisher WD, Laurin CA (1987). Rate of mortality for elderly patients after fracture of the hip in the 1980’s. J Bone Joint Surg Am.

[30] Magaziner J, Simonsick EM, Kashner TM, Hebel JR, Kenzora JE (1989). Survival experience of aged hip fracture patients. Am J Public Health.

[31] Bliuc D, Nguyen ND, Milch VE, Nguyen TV, Eisman JA, Center JR (2009). Mortality risk associated with low-trauma osteoporotic fracture and subsequent fracture in men and women. JAMA.

[32] Hu F, Jiang C, Shen J, Tang P, Wang Y (2012). Preoperative predictors for mortality following hip fracture surgery: a systematic review and meta-analysis. Injury.

[33] Pedregosa F, Varoquaux G, Gramfort A, Michel V, Thirion B (2011). Scikit-learn: Machine Learning in Python. J Mach Learn Res.

[34] Bergstra J, Bengio Y (2012). Random Search for Hyper-Parameter Optimization. J Mach Learn Res.

[35] Lundberg SM, Lee SI. A unified approach to interpreting model predictions. In: Proceedings of the 31st International Conference on Neural Information Processing Systems. 2017. https://papers.nips.cc/paper/2017/file/8a20a8621978632d76c43dfd28b67767-Paper.pdf. Accessed 1 May 2022.

[36] Lundberg SM, Erion G, Chen H, DeGrave A, Prutkin JM, Nair B (2020). From Local Explanations to Global Understanding with Explainable AI for Trees. Nat Mach Intell.

[37] Roberts SE, Goldacre MJ (2003). Time trends and demography of mortality after fractured neck of femur in an English population, 1968–98: database study. BMJ.

[38] Roche JJ, Wenn RT, Sahota O, Moran CG (2005). Effect of comorbidities and postoperative complications on mortality after hip fracture in elderly people: prospective observational cohort study. BMJ.

[39] Handoll HH, Farrar MJ, McBirnie J, Tytherleigh-Strong G, Milne AA, Gillespie WJ. Heparin, low molecular weight heparin and physical methods for preventing deep vein thrombosis and pulmonary embolism following surgery for hip fractures. Cochrane Database Syst Rev. 2002:Cd000305. 10.1002/14651858.cd000305. 10.1002/14651858.CD000305 12519540

[40] Castronuovo E, Pezzotti P, Franzo A, Di Lallo D, Guasticchi G (2011). Early and late mortality in elderly patients after hip fracture: a cohort study using administrative health databases in the Lazio region. Italy BMC Geriatr.

[41] Panula J, Pihlajamäki H, Mattila VM, Jaatinen P, Vahlberg T, Aarnio P (2011). Mortality and cause of death in hip fracture patients aged 65 or older: a population-based study. BMC Musculoskelet Disord.

[42] Breiman L (2001). Random Forests. Mach Learn.

[43] Natekin A, Knoll A (2013). Gradient boosting machines, a tutorial. Front Neurorobot.

[44] Tanphiriyakun T, Rojanasthien S, Khumrin P (2021). Bone mineral density response prediction following osteoporosis treatment using machine learning to aid personalized therapy. Sci Rep.

[45] Malmgen H, Borga M, Niklasson L (2000). Artificial Neural Networks in Medicine and Biology.

[46] Ottenbacher KJ, Linn RT, Smith PM, Illig SB, Mancuso M, Granger CV (2004). Comparison of logistic regression and neural network analysis applied to predicting living setting after hip fracture. Ann Epidemiol.

[47] Tu JV (1996). Advantages and disadvantages of using artificial neural networks versus logistic regression for predicting medical outcomes. J Clin Epidemiol.

[48] Dreiseitl S, Ohno-Machado L (2002). Logistic regression and artificial neural network classification models: a methodology review. J Biomed Inform.

[49] Terrin N, Schmid CH, Griffith JL, D’Agostino RB, Selker HP (2003). External validity of predictive models: a comparison of logistic regression, classification trees, and neural networks. J Clin Epidemiol.

[50] Sargent DJ (2001). Comparison of artificial neural networks with other statistical approaches: results from medical data sets. Cancer.

[51] Work JW, Ferguson JG, Diamond GA (1989). Limitations of a conventional logistic regression model based on left ventricular ejection fraction in predicting coronary events after myocardial infarction. Am J Cardiol.

[52] Webb GI, Sammut C, Webb GI (2010). Naive Bayes. Encyclopedia of Machine Learning.

[53] Ng AY, Jordan MI. On discriminative vs. generative classifiers: a comparison of logistic regression and naive Bayes. In: Proceedings of the 14th International Conference on Neural Information Processing Systems. Natural and Synthetic; 2001. https://proceedings.neurips.cc/paper/2001/file/7b7a53e239400a13bd6be6c91c4f6c4e-Paper.pdf. Accessed 1 May 2022.

[54] Vapnik V (2000). The Nature of Statistical Learning Theory.

[55] Raghavendra NS, Deka PC (2014). Support vector machine applications in the field of hydrology: a review. Appl Soft Comput.

[56] Zhang XH, Heller KA, Hefter I, Leslie CS, Chasin LA (2003). Sequence information for the splicing of human pre-mRNA identified by support vector machine classification. Genome Res.

[57] VanBelle V, Lisboa P. Research directions in interpretable machine learning models. In: Proceedings of the European Symposium on Artificial Neural Networks, Computational Intelligence and Machine Learning (ESANN2013). 2013. https://citeseerx.ist.psu.edu/viewdoc/download;jsessionid=9481C4C77A4FBFC1E97A75E09DAC5715?doi=10.1.1.642.9731&rep=rep1&type=pdf. Accessed 1 May 2022.

[58] Zhang Z. Introduction to machine learning: k-nearest neighbors. Ann Transl Med. 2016;4:218. 10.21037/atm.2016.03.37. 10.21037/atm.2016.03.37 PMC4916348 27386492

[59] Islam MJ, Wu QMJ, Ahmadi M, Sid-Ahmed MA. Investigating the performance of Naive-Bayes Classifiers and K-Nearest Neighbor Classifiers. J Converg Inf Technol. 2010. 10.4156/jcit.vol5.issue2.15.

[60] Zhang Z (2014). Too much covariates in a multivariable model may cause the problem of overfitting. J Thorac Dis.

[61] Amarasingham R, Patzer RE, Huesch M, Nguyen NQ, Xie B (2014). Implementing electronic health care predictive analytics: considerations and challenges. Health Aff (Millwood).

[62] Charlson ME, Pompei P, Ales KL, MacKenzie CR (1987). A new method of classifying prognostic comorbidity in longitudinal studies: development and validation. J Chronic Dis.

[63] Jou HJ, Siao RY, Tsai YS, Chen YT, Li CY, Chen CC (2014). Postdischarge rehospitalization and in-hospital mortality among Taiwanese women with hip fracture. Taiwan J Obstet Gynecol.

[64] Downey C, Kelly M, Quinlan JF (2019). Changing trends in the mortality rate at 1-year post hip fracture—a systematic review. World J Orthop.

[65] Helm JM, Swiergosz AM, Haeberle HS, Karnuta JM, Schaffer JL, Krebs VE (2020). Machine Learning and Artificial Intelligence: Definitions, Applications, and Future Directions. Curr Rev Musculoskelet Med.

[66] Li Y, Chen M, Lv H, Yin P, Zhang L, Tang P (2020). A novel machine-learning algorithm for predicting mortality risk after hip fracture surgery. Injury.

[67] Colón-Emeric CS, Saag KG (2006). Osteoporotic fractures in older adults. Best Pract Res Clin Rheumatol.

[68] Miller BJ, Callaghan JJ, Cram P, Karam M, Marsh JL, Noiseux NO (2014). Changing trends in the treatment of femoral neck fractures: a review of the american board of orthopaedic surgery database. J Bone Joint Surg Am.

[69] Kim SJ, Park HS, Lee DW (2020). Outcome of nonoperative treatment for hip fractures in elderly patients: A systematic review of recent literature. J Orthop Surg (Hong Kong).

[70] Vidal E, Moreira-Filho D, Pinheiro R, Souza RC, Almeida L, Camargo K (2012). Delay from fracture to hospital admission: a new risk factor for hip fracture mortality?. Osteoporos Int.

