# Supplementary material for: Development and internal validation of a machine-learning-developed model for predicting 1-year mortality after fragility hip fracture
Source: BMC Geriatr. 2022 May 24;22:451. doi: 10.1186/s12877-022-03152-x (PMC9131628; doi:10.1186/s12877-022-03152-x)
Supplement: Supplementary file 1 — Additional file 1. [file 12877_2022_3152_MOESM1_ESM.pdf]

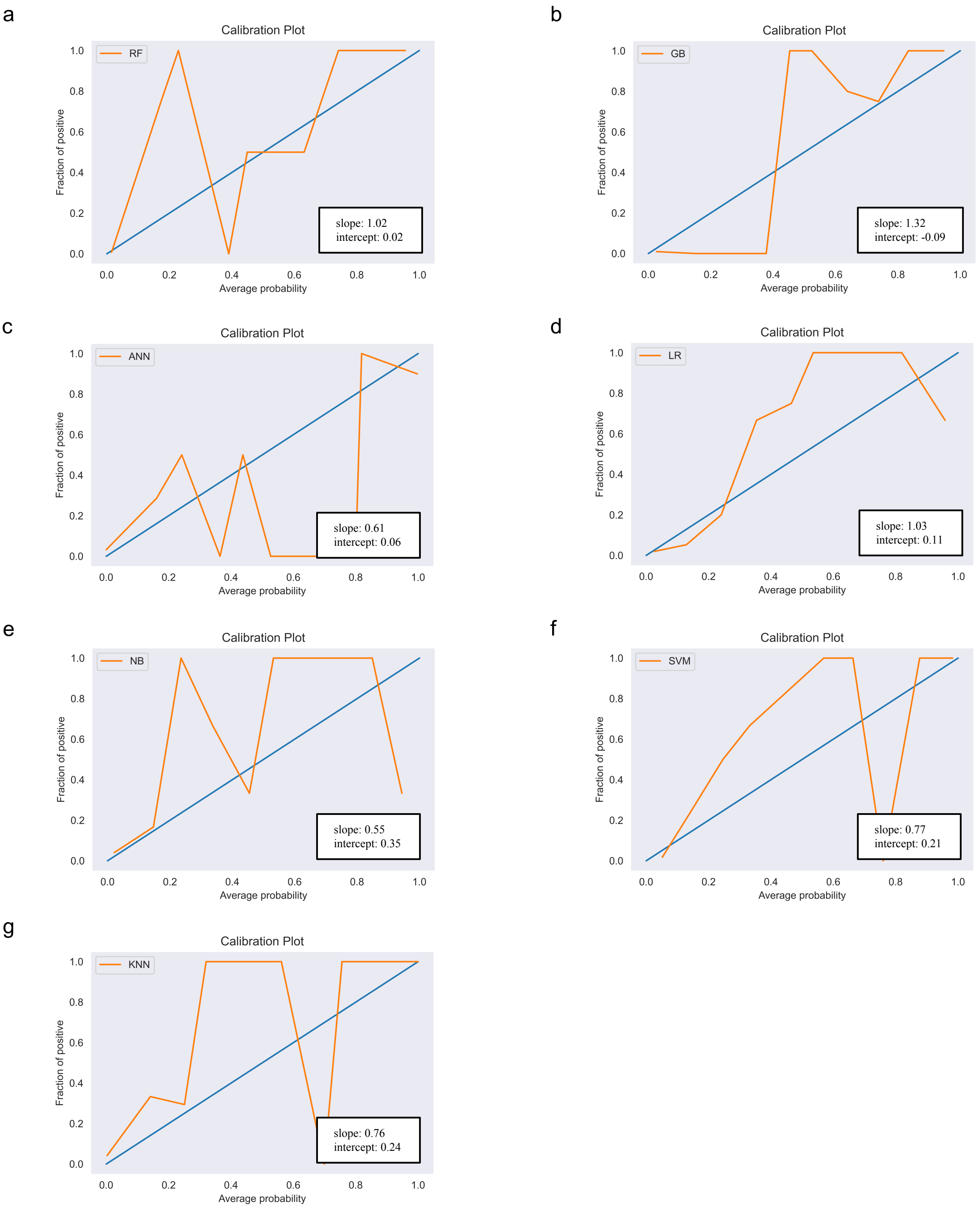

Supplementary file 1. Calibration plot of **(a)** Random Forests algorithm (RF); **(b)** Gradient Boosting algorithm (GB); **(c)** Artificial Neural Network algorithm (ANN); **(d)** Logistic Regression algorithm (LR); **(e)** Naive Bayes algorithm (NB); **(f)** Support Vector Machine algorithm (SVM); and **(g)** K-Nearest Neighbors algorithm (KNN).
